# When choice is not performative: the struggle for agency among women accessing family planning services in Senegal

**DOI:** 10.3389/fgwh.2026.1826792

**Published:** 2026-08-26

**Authors:** Thais Gonzalez-Capella, Marieme Fall, Mbenoye Ndiaye, Cheikh Issa Diop, Manuela Reveiz, Nour Horanieh, Elizabeth Larson, Beniamino Cislaghi

**Affiliations:** 1 Department of Global Health and Development, London School of Hygiene and Tropical Medicine, London, United Kingdom; 2 Ideas42, Dakar, Senegal; 3 Université Alioune Diop de Bambey, Bambey, Senegal; 4 Université Cheikh Anta Diop, Dakar, Senegal; 5 School of Population and Global Health, Faculty of Medicine and Health Sciences, McGill University, Montreal, QC, Canada; 6 Department of Family and Community Medicine, College of Medicine, King Saud University, Riyadh, Saudi Arabia; 7 Center on Gender Equity and Health, University of California San Diego, San Diego, CA, United States; 8 School of Social Work, Makerere University, Kampala, Uganda; 9 Institute for Development Studies, Brighton, United Kingdom

**Keywords:** agency, coercion, contraception, ethics, family planning, informed choice

## Abstract

**Introduction:**

Despite ongoing efforts to improve family planning (FP) counselling as a means of increasing uptake and safeguarding women's sexual and reproductive rights, interactions between FP providers and users continue to play a decisive role in shaping contraceptive decisions. In practice, these encounters may influence method choice in ways that raise questions about the extent of women's reproductive autonomy. Drawing on theoretical perspectives on agency and informed choice, this study examines how women in Senegal navigate counselling and enact agency in FP decision-making.

**Methods:**

This qualitative study included 256 participants (130 men and 126 women) from four Senegalese regions: Dakar, Tambacounda, Ziguinchor, and Matam. Data were collected through 116 semi-structured interviews and 16 focus group discussions (8–10 participants per group), using purposive sampling. Discussions explored experiences related to FP service delivery and data were analyzed thematically.

**Results:**

Findings suggest that participants perceived some counselling practices as encouraging the adoption of LARCs in ways that limited their ability to make autonomous contraceptive decisions and influenced method choice during client-provider counselling interactions in Senegal. Women described a range of counselling practices from providers, such as presenting the implant as the primary or only available method, selectively emphasizing or omitting side-effects, limiting opportunities for dialogue, or expressing resistance to implant removal. In response to these dynamics, some women reported choosing to withdraw from contraception altogether. Such decisions may reflect constrained circumstances in which exercising agency may come at the expense of women's reproductive intentions and the right to effectively manage the timing and spacing of their own pregnancies.

**Discussion:**

Safeguarding freedom of choice, and the ability to act upon that choice is a central but complex objective of FP programming in Francophone West Africa (FWA). Our findings underscore the pressing need for improved policy dialogue and ethical reflection on FP service delivery practices, more focused on women's lived experiences, including their expressions of hesitation, fear, resistance, or withdrawal, which are essential to understanding how agency is enacted in practice and to strengthening rights-based approaches to family planning across contexts.

**Conclusion:**

Based on the experience from 256 adult women and men of reproductive age in Senegal, this study demonstrates that method choice unfolds within complex normative and institutional landscapes, where counselling practices can either enable or constrain women's reproductive agency, underscoring the urgent need to strengthen rights-based, autonomy-centered family planning approaches.

## 1 Introduction

Respecting users’ choices is at the center of Family Planning (FP) interventions, but few address the complexity of determining whether women are truly making informed choices rather than being influenced, persuaded or coerced (1). FP counselling services are of utmost importance as they influence the process of making decisions about contraception and choosing appropriate methods, which can potentially limit users’ agency and contraceptive autonomy (2). Informed choice is defined as the right of individuals or couples to make a voluntary, well-considered decision or a choice that is based on options, information, and understanding (3). Ensuring informed decision-making is particularly crucial for the adoption of long-acting reversible contraceptives (LARCs), such as implants or intrauterine devices (IUDs), because their removal requires the assistance of a trained provider (4). Research has shown that clients who have not been appropriately counselled on the characteristics of their chosen method, including how it works, its potential side effects, and their management, are more likely to discontinue contraception method within the first year (5).

An expanding body of evidence is calling into question the prevailing assumption that the promotion of modern contraceptives necessarily entails women's empowerment, as it often presumes that the uptake reflects informed choice and is therefore free of coercion (5–7). Access to family planning services is essential for women's reproductive health and rights, and the reduction of maternal and child mortality. Yet, the quality of interactions within FP counselling services, remains a critical and often overlooked determinant of whether contraceptive choices genuinely reflect women's preferences (8).

Back in 1990, a quality-of-care framework identified “choice of methods”, “the information given to clients”, and “interpersonal relations” as essential elements of respectful family planning programmes (9). As long-acting reversible contraceptives (LARCs) uptake proliferates in Francophone West Africa (FWA), concerns around the potential lack of information, trust, and social network pressure influencing women's contraceptive choices are increasing within the international FP community (10). With advances in modern contraceptive technologies during the last decades, women across different settings have reported being influenced towards the use of LARCs, describing feelings of frustration when they perceived that their preferences were overlooked in contraceptive method choice decision-making (5, 11).

Women's agency refers to their ability to act on their choices and goals, exercise their individual autonomy, and make informed decisions regarding their reproductive health (12). Agency encompasses their capacity to freely choose, and access contraceptive methods based on accurate information, personal preferences, and reproductive desires. Women's agency in family planning involves the recognition of their rights to control their own bodies, determine the timing and spacing of pregnancies, and actively participate in decision-making processes without coercion or external influences (13). It emphasizes the importance of empowering women to have control over their reproductive lives and promoting their overall well-being and reproductive rights (11, 12).

In this study, we conceptualize agency as the situated capacity to navigate reproductive decision-making within unequal social and institutional conditions (1, 14). Actions such as refusal, discontinuation, or disengagement from services may sometimes reflect attempts to assert preferences under constrained circumstances, while also being shaped by fear, uncertainty, misinformation, or structural limitations. Distinguishing between autonomous choice and constrained decision-making is therefore central to interpreting women's experiences within FP counselling interactions.

Several studies suggest that women in Francophone West Africa may lack information about family planning options or may not feel comfortable discussing these issues with health care providers (15). This can lead to a lack of agency in decision-making and limited access to services. Overall, these discussions highlight the need for a holistic approach to promoting women's agency and autonomy in relation to family planning services in FWA and elsewhere. This includes addressing social and cultural norms, improving provider training and accountability, and promoting women's knowledge and confidence in making informed decisions about their reproductive health (14).

Nine countries in Francophone West Africa (Benin, Burkina Faso, Guinea, Ivory Coast, Mali, Mauritania, Niger, Senegal, and Togo) are members of the Ouagadougou Partnership, which aims to double the number of users of modern contraceptive methods to 13 million by 2030, adding 6.5 million users (16). Senegal was selected as a case-study due to its strong national prioritization of family planning to expand contraceptive options (17). Between 2011 and 2023, the country's modern contraceptive prevalence rate increased from 13.1% to 26.5%, with implants as the most used method (42%), followed by injectables (30.7%) and pills (13.6%) (18). This preference for implants is notable given that 43.3% of women in reproductive age wish to space births for two years or more, while only 18.7% wish to stop childbearing (18), highlighting the need to examine social and programmatic factors influencing contraceptive choice in Senegal.

## 2 Materials and methods

### 2.1 Study settings

Senegal is a lower-middle-income West African nation that has maintained democratic continuity since its 1960 independence from France, despite political volatility surrounding the 2024 elections (19). While the country leads globally in legislative parity, with women holding 46.1% of parliamentary seats, female autonomy remains restricted by patriarchal customary frameworks and limited female representation in executive leadership, particularly regarding land inheritance and reproductive decision-making (20). This social landscape is further defined by a significant youth bulge, with 60% of the 18.7 million inhabitants under age 25, and distinct ethnic distributions: *Wolof* (52.8%) and *Sereer* (9.7%) groups are primarily urban based, while *Pulaar* (26.7%) and *Mandinka/Soninke* groups reside largely in northern and southern border zones (21). These regional divides correlate with a spatially structured health inequalities: although the national maternal mortality ratio has improved to 153 per 100,000 live births, it remains far from SDG 3 target of 70 per 100,000 live births, with peripheral regions like Tambacounda facing much higher risks (up to 284 deaths) due to geographic isolation (18).

Evidence suggests that progress in the health sector is driven by skilled birth attendance, which rose to 77% in 2020, and community initiatives like the *Badiénou Gox* (women caregivers or community mediators), yet deep socioeconomic gaps persist. Modern contraceptive use has reached 24.1%, while the unmet need for family planning stands at 14.4% (18). Although 69% of health facilities nationwide offer FP services, there are notable variations among regions (22). Health centers are the facilities in which all methods are more frequently offered. The contraceptive prevalence rate (CPR) in regions such as Tambacounda and Matam is largely below the national average (<15%), whereas in Ziguinchor it is between 19% and 24%, and Dakar has at CPR above the national average (> 38%) (18).

### 2.2 Sampling and data collection

To capture insights from women and men exposed to family planning (FP) programs in Senegal, data were collected across four selected regions representing diverse socio-demographic contexts: urban Dakar, suburban Ziguinchor, rural Matam, and peri-urban Tambacounda. A total of 256 participants aged 18 years and older were included in the study, comprising 116 semi-structured individual interviews (SI) and 16 focus group discussions (FGDs). The sample included women (*n* = 126) and young adults aged 18–25 years (*n* = 118). The FGDs involved 140 individuals and were stratified by age group (18–25 years and >25 years) and gender (young women, young men, older women, and older men) to facilitate open discussion and encourage participants to speak freely about sensitive topics. The 25-year cutoff was selected to reflect differences in reproductive trajectories, partnership dynamics, and social expectations surrounding fertility and contraceptive use between younger and older participants.

Participants were recruited using purposive sampling with support of community workers and local leaders. Eligible participants included women and men who were current users of family planning services or had accessed such services in Senegal within the previous three years. As some of these actors were connected to local health and social networks, their involvement may have introduced selection bias by increasing the likelihood of recruiting individuals more familiar with family planning services than the broader community population. All interviews and FGDs were conducted in private settings, and interviewer participant gender concordance was ensured whenever possible.

Fieldwork was conducted by sixteen Senegalese researchers experienced in qualitative research and familiar with the study regions. Topic guides were informed by a scoping review on ethical challenges among FP interventions in Sub-Saharan Africa (23), and refined collaboratively by the research team and locally based researchers to ensure contextual relevance. Training included role-playing and interactive sessions to strengthen interviewing skills and ethical reflexivity. Tools were piloted in Dakar and revised accordingly. Following ethical approvals, four data collectors were assigned to each region based on linguistic competencies and local knowledge.

After obtaining verbal and written informed consent, interviews and FDGs were conducted and recorded in French, *Wolof*, *Pulaar*, or *Jola*. Recordings were translated and transcribed into French by trained Senegalese researchers to enable collaborative analysis. Individual interviews lasted approximately one hour, while FGDs lasted around three hours with a break with refreshments provided. To ensure confidentiality while maintaining analytic transparency, participants were assigned anonymized codes indicating gender and age category (YW = young woman aged 18–25; OW = older woman aged >25 years; YM = young man aged 18–25 years; OM = older man aged >25 years), followed by a sequential number, and the region (DK, ZG, MT, TB).

### 2.3 Data analysis

Thematic analysis, an iterative process of identifying and interpretating patterns within qualitative data (24, 25) was supported by NVivo V. Pro Software for data management and coding. An initial subset of transcripts was open coded by one researcher to develop a preliminary coding framework. The framework was then applied and independently tested through blind coding by multiple team members, with codes iteratively refined through comparison and discussion until consensus was achieved. Researchers subsequently coded data across demographic groups and regions. As analysis progressed, the coding framework was revised to incorporate emerging themes until saturation was reached. A participatory interpretation workshop in Dakar with seven original data collectors facilitated collaborative axial coding and refinement of key analytical themes (26). Subsequently, joint writing workshops were held in Dakar to finalize the analysis and prepare the manuscripts.

## 3 Results

Exploring the perspectives of family planning users revealed complexities within interactions between family planning users and providers, revealing power asymmetries that shape both decision-making and users’ experiences of FP counselling. Two central themes emerged from the analysis. First, older and young women, described experiencing pressure from providers to adopt long-acting reversible contraceptives (LARCs), suggesting that provider preferences often played a decisive role in shaping method choice. Participants further perceived that providers did not always communicate information in a comprehensive or transparent manner, potentially limiting informed decision-making and user autonomy. Second, several women reported opting out of contraception altogether, when faced with fear or uncertainty. Such decisions can be understood as expressions of agency enacted through refusal or disengagement, yet they also represent missed opportunities to address unmet family planning needs. Together, these themes shed light on the structural and interpersonal factors that shape family planning experiences, with implications for both service delivery and policy design.

### 3.1 Theme 1—constrained agency and provider-driven pressure towards implants

Our findings reveal a recurrent pattern in which certain family planning (FP) providers appeared to promote specific long-acting reversible contraceptives (LARC), particularly implants, which were implicitly or explicitly prioritized over other methods, often at the expense of women's expressed preferences for short-acting methods. This tendency was observed across all four regions and for women of all ages. Rather than presenting a balanced range of options, some user-provider interactions reflected counselling practices that narrowed women's decision-making space and redirected their choices toward implants.

Several recurring counselling practices that appeared to limit women’s agency in contraceptive method selection were described by study participants:

1. Implants presented as the primary or only option

Several women reported that during FP counselling sessions, certain providers presented the implant as the only or relevant method, limiting comparative deliberation. A young woman in Ziguinchor explained:

“I once heard about the injection method. This was before I was doing family planning. But when I was in front of the midwife, she didn't show me that method. As I told you, she showed me the implant. She didn't mention this method. She only showed me the implant.” (YW-9, ZG)

Likewise, another young woman in Tambacounda:

“Q: Did they show you the injection or any other method like pills or something?

A: No, they only had the implant with them” (YW-3, TB)

When counselling is structured around one single method, women’s agency is shaped by the limitation of options during her decision-making process.

2. Framing women's “forgetfulness” as a justification for implants

Additional mechanisms were also reported as constraining women's agency in method choice during FP counselling. For example, a young woman for rural Matam reported being discouraged from choosing the three-month injection on the grounds that she might forget subsequent medical appointments:

“Q: Did they tell you about birth control methods before you chose?

A: No, they just told me that with the three-month method, I could forget my appointments” (YW-5, MT)

While framed as a practical adherence concern, this rationale positions women as unreliable users, while somehow legitimizing the implant as a more “suitable” option. Once again, the provider narrowed autonomous deliberation of the woman.

3. Selective emphasis and omission of side-effects

Several accounts suggested how providers emphasized potential side effects of short-acting methods while omitting comparable discussion on implant-related side effects. For instance, an older woman in Matam described how the FP provider emphasized the potential side-effects of her preferred short-acting method in a way that appeared to direct her towards the long-acting implant.

“I informed her that I wanted to use the injection, and she told me that the injection was not safe because you don't know the consequences behind it. She offered me the implant which I finally chose. She just explained the consequences of the injection because she didn't want me to use it” (AW-6, MT)

In a similar account, when an older woman in urban Dakar expressed her preference for the injection, the FP provider cited “feet ache” as a potential side effect, while this symptom is not listed in the WHO Manual for Family Planning providers (WHO, 2022), and no comparable consequences were discussed about the implant:

“I told her that I wanted to use the injection, but she asked me to choose the implant since I wanted to do family planning for just one year. She told me that it was a risk to take an injection every 3 months. According to her, when you take the injection, it can cause you to have a feet ache” (OW-6, DK)

When she further sought additional reassurance at the pharmacy, staff advised her to follow the midwife’s recommendations:

“There were no power moves because she told me to choose the implant. However, I went to ask the lady in the pharmacy to clarify the method that the midwife had suggested, and she suggested that I do it since that's what the midwife told me” (AW- 6, DK)

Such counselling practices described throughout this section, suggest a dynamic in which women's stated preferences may be redirected through selective presentation of side effects that favour the uptake LARCs, particularly the implant. Rather than supporting informed and autonomous decision-making, these interactions appear to limit the woman's agency by constraining her method choice. The sequence of interactions shows how efforts to seek a second opinion did not expand her decision-making space but rather reinforced institutional authority. These accounts illustrate counselling dynamics experienced by participants as limiting autonomous contraceptive decision-making, potentially disregarding women's preferences and reproductive rights.

4. Limited opportunity for dialogue:

Several older women in Dakar also described one-directional interactions in which questions were discouraged or unanswered. An older woman in Dakar mentioned:

“They didn't explain the advantages or disadvantages of the method you choose (..). After I made my choice, they just put it in, and I left. But I wasn't comfortable (…) because I was doing it without really knowing the consequences. They didn't explain anything about the pros and cons; they just showed me the methods and then I made my choice. I didn't ask them any questions because I didn't feel I could.” (OW-1, DK)

Likewise, another woman explained her experience on how the FP provider did not respond to her questions and concerns:

“She says what she has to say and then it's over. And when you ask her questions, she doesn't even answer you. (..) I didn't have the opportunity to ask her questions because she was the only one talking. Sometimes I would ask her questions, but she wouldn't answer me.” (OW-7, DK)

Another woman in Dakar in a similar way explained how she was not informed about potential side effects and did not have the opportunity to ask about them to the FP provider:

“I only had to make my choice when she explained the family planning methods, but I didn't have the opportunity to ask her about the consequences.” (OW- 8, DK)

During these encounters, women's agency appears to be constrained by the lack of space for dialogue and exchange with providers. Interestingly, these accounts are primarily articulated by older women, framed as explicit complaints about not being heard. In contrast, younger women did not describe interactions in similarly critical terms. These generational differences may point to potential variations in how agency is perceived and articulated when navigating clinical authority by users across ages.

Interestingly, several men also articulated opinions about the lack of communication between providers and female users. A male informant in urban Dakar, expressed trust in family planning services and acknowledged their intended benefits to improve family's wellbeing. At the same time, he also identified shortcomings in provider-client communication, suggesting that confidence in family planning services may coexist with concerns about inadequate information provision that could compromise the effectiveness of FP services:

“A: No, no, it's still there because every day they educate people to practice family planning. But it remains a lot because the communication is insufficient. We need much more communication.

E: What do you think they [FP providers] are looking for?

A: That every family has a peaceful life, good health, children and a mother. Because managing families is very difficult right now.” (AM-2, DK)

Another man in Ziguinchor, clearly defended the right of women as FP services users to have access to all the information related to advantages and disadvantages of contraceptive methods, to support the woman in decision-making for the most suitable method according to her needs:

“There, I think their health facility is obligated to give all the necessary information related to the different contraceptive methods: the pros and cons, the risks they face as well and all that. Now, right now, if the woman is not well informed; if she's not very knowledgeable about the pros and cons, she can be steered into making a choice that's not right for her.” (OM- 4, ZG)

5. Resistance to method removal

Furthermore, one woman from urban Dakar described encountering resistance from FP providers when requesting the removal of her implant, a concern she suggested was shared by other women. Notably, although our interview topic guide did not explicitly include specific questions on barriers to LARC removal, this issue emerged spontaneously during the interview. She expressed frustration with long waiting times for implant removal and perceived that women requesting discontinuation were given lower priority than new contraceptive users by family planning providers:

“One time when I wanted to remove my contraceptive method, I heard them asking questions about why do people want to remove family planning when the opposite should be happening? I take it that the fact that women want to take away planning is not convenient for them since there were 6 of us who wanted to do it. Everyone is trying to give their reasons for wanting to remove it. They told us to wait so they could finish with those who wanted to do family planning first before finishing with us. The providers didn't want us to remove it.” (AW-1, DK)

This account underscores that agency in FP extends beyond the initial choice of a contraceptive method, to include the ability to discontinue its use when it no longer aligns with a woman's reproductive preferences. For methods requiring provider-assisted removal, such as implants, exercising this dimension of agency depends on timely access to professional support. Barriers to removal may therefore constrain women's reproductive autonomy by preventing women from acting upon their evolving reproductive preferences.

### 3.2 Theme 2—agency enacted through negotiation and refusal

The notion of Agency is central to ethical family planning (FP) service provision, particularly in contexts where reproductive decision-making is shaped by restrictive gender norms, such as the case of Senegal. Our data shows how women navigate these norms within interactions with healthcare providers by either resisting, negotiating or even withdrawing from services altogether.

In one instance, an older woman from the peri urban region of Tambacounda explained having to persuade the provider to respect her decision to use contraception without her husband's consent. Although the provider initially questioned her decision, the woman was able to assert her reproductive preferences and ultimately secure access to contraception:

“ Before she [FP provider] started explaining, she did not agree that I had made the decision without my husband's consent. But when I explained it to her, she finally understood. I told her that it was not a matter of not wanting to have children, but that I'm going through a complicated situation and thinking about the future of my children.” (OW-1, TB)

This example illustrates agency as a negotiation within socially entrenched power structures. From an ethical standpoint, FP services should not require women to justify or defend their reproductive decisions. However, this account suggests that access to contraception may remain contingent to women's ability to negotiate with providers rather than on the straightforward recognition of their reproductive preferences.

In contrast, another young woman in rural Matam responded differently when confronted with a provider who did not acknowledge her contraceptive method preferences. In the following statement, the participant's refusal to use contraception may be understood as an attempt to exercise agency within constrained counselling conditions. This decision appeared to be shaped by limited alternatives, and dissatisfaction with the counselling encounter:

“I wanted to do the three-month injection, but the provider asked me to do a five-year implant. I didn't want that method, and she wouldn't give me the method of my choice. In the end, I didn't do family planning” (YW-2, MT)

This outcome highlights an ethical paradox in FP programming, suggesting that provider pressure may produce the opposite effect to that intended. Rather than increasing contraceptive uptake, pressure can lead to mistrust and discourage the user to use any method at all, ultimately reinforcing the unmet need for family planning.

A related dynamic emerged in the account of an older woman in Dakar who had chosen the contraceptive pill and obtained it from the provider, but later decided not to use it due to insufficient information:

“I had decided to choose the pill. When I got home, I couldn't use it because I was afraid. I didn't have enough information that would make me use it” (OW-7, DK)

Within this interaction, agency was exercised through withdrawal in response to uncertainty and fear, reflecting a constrained form of agency rather than fully autonomous decision-making. The absence of adequate counselling on the chosen method limited her confidence, ultimately leading to non-use despite her initial choice.

An older man in Tambacounda offered a different perspective. In his view, providing detailed information about the health implications and potential side effects of contraception could generate fear and ultimately discourage its use:

“Yes, because the midwives discuss with them. Once telling them that it [contraception] avoids early pregnancies at the same time improves their health, that it spaces and limits the births and has direct consequences especially on their health. If we tell them all this, they will be afraid and will back off. They won't do it.” (OM-2, TB)

His account reflects ambivalence toward comprehensive counselling. While information is intended to support choice, this informant suggests that “too many” explanations may overwhelm women, leading them to not use contraception. Once again, such withdrawal can also be interpreted and an expression of agency: when confronted with perceived risk or uncertainty, women may choose not to use contraception.

However, if these women were to seek counselling because their desire is to address a reproductive need, disengagement following a “comprehensive” counselling encounter may represent a lost opportunity to respond to that need. Together, these narratives illustrate how contraceptive autonomy requires not only access to FP services but also balanced and tailored information and respect for women's expressed reproductive preferences.

## 4 Discussion

The results of this study explore the interactions between the family planning providers and the users of the services in Senegal. We intend to increase the understanding of what lies beneath the choice of a woman regarding the use of a contraceptive method and the level of agency FP users may have when interacting with FP providers.

Rights-based family planning care is influenced not only by access to services and informed decision-making, but also by wider socio-cultural factors such as gender norms, power relations, stigma, provider behaviors, and community expectations that shape reproductive agency and contraceptive choices. However, our study focused primarily on informed choice and counselling dynamics because these themes emerged most strongly from participants’ narratives and aligned with the specific objectives of the qualitative inquiry (27, 28).

Contemporary discussions continue to foreground “informed choice”, typically assessed on whether users receive information about the full range of methods, their potential side effects, and the possibility of switching methods if desired. However, “informed choice” primarily captures whether information is provided and not necessarily whether individuals are able to act upon their preferences (1). The capacity to translate preference into action, often conceptualized as autonomy or agency, requires more than information. Our findings suggest that the ways in which user-provider interactions shape reproductive agency are often subtle, complex, and not always easily perceptible.

Participants described a range of experiences in contraceptive decision-making. Some reported selecting and obtaining their preferred contraceptive method without perceiving external pressure or provider influence. Others described trusting their healthcare provider and accepting professional recommendations, often without seeking additional information or clarification. Despite these different decision-making processes, participants generally regarded the choice as their own rather than as one imposed by providers.

Within FP discourse, coercion is often understood in its most extreme forms, such as non-consensual sterilization or denial of abortion services. Yet emerging evidence, including findings from this study, indicates that more subtle dynamics, such as provider pressure to adopt certain methods, may be more subtle but common (1). These forms of influence operate within routine counselling interactions and can affect women's decision-making even when formal consent procedures are followed. In fact, our data showed how several providers would encourage women to use LARCS for many different reasons.

The predominance of implant-related narratives in our findings reflects the empirical material generated during interviews, rather than an intentional exclusion of other LARC methods such as Intrauterine Devices (IUDs). For example, some participants described counselling interactions in which greater emphasis was placed on the potential side effects of short-term methods, including effects not consistently reflected in national family planning guidance, while comparatively less discussion was reportedly dedicated to the possible side effects associated with LARCs such as implants.

Current evidence suggests several possible rationales underlying pressure to adopt LARCs. Some scholars have argued that provider pressure toward LARCs may, in certain contexts, be shaped by broader demographic targets, institutional incentives, or quantitative performance indicators within family planning programs (29). However, our data do not allow us to directly assess the motivations driving providers’ counselling practices in Senegal. Our conclusions should therefore be understood as heuristic and interpretive, rather than ultimate and definitive.

Despite providers intentions to uphold informed choice and client autonomy, structural and contextual constraints may shape counseling practices and contribute to provider bias, as documented in a systematic review of contraceptive counseling in Western settings (30). In contexts where providers work closely with vulnerable populations, recommendations for LARCs may reflect efforts to protect women's health by preventing high-risk pregnancies rather than an intention to reduce population growth (2, 31).

In other cases, participants also described instances in which providers refuse to remove LARCs. As illustrated in the Results section, one woman described how providers prioritized contraceptive initiation over method removal, even when women reported side effects or expressed a desire to discontinue contraception. Similar findings have increasingly been documented in recent literature (11, 32). User's accounts reveal diverse perceptions of providers and highlighted communication challenges. These ranged from the fear of asking further questions about a contraceptive method to a sense that relevant information had not been fully disclosed. Although some participants viewed providers as well intentioned and concerned about women and couples’ well-being, they nevertheless acknowledged the need for clearer and more comprehensive communication.

Discontinuation or withdrawal from a contraceptive method can constitute an expression of women's reproductive agency and autonomy in managing their fertility preferences. However, such decisions may also reflect inadequate counselling on method management, side effects, or switching options prior to adoption. In Francophone West Africa, debates about women's agency in family planning services frequently emphasize the influence restrictive gender and cultural norms (33). Scholars argue that cultural and religious beliefs, and entrenched gender roles may limit women's ability to make independent reproductive decisions, while the use of family planning may be stigmatized within families and communities (10). However, emerging scholarship suggests that certain FP promotion strategies may reproduce more subtle constraints on women's agency. For instance, a critical visual discourse analysis of long-acting reversible contraception (LARC) promotion materials argues that contemporary LARC imagery constructs what the authors term as “agency-without-choice”, where LARC methods are framed as the only responsible contraceptive option, ironically justified through narratives of empowerment (34).

Men's narratives further revealed ambivalent positioning within FP counselling dynamics. While several male participants expressed trust in family planning services and recognized their potential benefits for family wellbeing, they simultaneously articulated concerns regarding insufficient communication, women's lack of information, lack of men's involvement, and the possibility that women could be directed toward methods that did not align with their preferences. These perspectives suggest that concerns regarding informed choice and counselling quality were not exclusively raised by women participants.

These counselling dynamics should also be understood within broader gendered systems of reproductive responsibility. Women were frequently positioned as responsible for managing fertility while simultaneously navigating provider authority, expectations surrounding motherhood, and, in some cases, the perceived need for male approval. Some provider recommendations appeared shaped not only by clinical considerations but also by gendered assumptions regarding women's adherence, reliability, and reproductive roles. In this sense, counselling interactions reflected broader power asymmetries embedded within reproductive health systems and social norms surrounding women's reproductive decision-making (5, 11, 32).

Further research is needed to better understand why many family planning (FP) users appear to follow providers’ recommendations without expressing disagreement, even when experiencing fear or hesitation. Future studies should also examine the structural, institutional and programmatic factors that may shape providers’ counselling practices, biases, and attitudes towards clients, and how these are influenced by the context.

Given the qualitative nature of this study, the findings are not intended to be statistically generalizable to broader populations. Nevertheless, the results provide insights into the everyday realities women navigate when engaging within family planning services in pursuit of their sexual and reproductive rights and intentions. These lived experiences highlight the utmost importance of aligning family planning programming and funding priorities with the ethical and relational dimensions of care and counselling as they are experienced in practice.

## 5 Conclusion

Using qualitative data gathered from 256 adult and young women and men of reproductive age (25–49 years) across four regions in Senegal, this study explored how Family Planning (FP) users exercise their agency for a free and informed choice of contraceptive methods. Our results showed how FP users experience provider-driven pressure towards use of LARCs, a lack of FP provider transparency from users, often not mentioning side effects of LARCs that they are directing women towards to.

This study acknowledges the important contribution of LARCs and other FP methods in advancing sexual and reproductive rights. At the same time, our findings identified less visible dynamics that may influence contraceptive decision-making, including counselling practices that imply subtle forms or pressure or coercion embedded within routine FP counselling encounters. Safeguarding freedom of choice, and the ability to act upon that choice is a central but complex objective of FP programming, which requires improved policy dialogue and ethical reflection on service delivery practices. By attending closely to users’ lived experiences, particularly women's, including their expressions of hesitation, fear, resistance or withdrawal, is essential for understanding how agency is enacted in practice for strengthening rights-based approaches to family planning.

Our data and findings lead us to recommend the immediate improvement of FP counselling services to provide more complete and thus transparent information about methods and side effects, mixed-methods research about logistics and availability of different contraceptive methods and building upon existing research for improved metrics of the success of these programs that goes beyond measurement of LARCs uptake.

### 5.1 Strengths and limitations

Although this study included male and female participants from diverse regions and age groups in Senegal, selection bias may have occurred during the recruitment process. As a multilingual qualitative study, additional bias might have arisen during translations across Wolof, Pulaar, Jola, French, and English. Researcher bias and social desirability bias should also be taken into consideration. Furthermore, the study did not include perspectives from adolescents, an essential demographic requiring targeted family planning services in Senegal and Francophone West Africa. To mitigate these biases, the study incorporated several strengths, such as pre-data collection training, triangulation of data through interviews and focus group discussions, thorough data analysis with member cross-checking, and collaborative interpretation by a culturally diverse team engaging in ongoing reflexivity. Our study also relied primarily on users’ narratives and did not include direct observation of counselling encounters or provider-focused interviews specifically examining these interactions, limiting the ability to triangulate participants’ interpretations of counselling dynamics. Finally, this article is also accessible in French, with the goal of fostering a more inclusive global dialogue on Ethics and SRHR, especially for SRHR professionals working in French-speaking contexts (35).

## Data Availability

The raw data supporting the conclusions of this article will be made available by the authors, without undue reservation.
